# TGF-β1 Mediates Novel-m0297-5p Targeting *WNT5A* to Participate in the Proliferation of Ovarian Granulosa Cells in Small-Tailed Han Sheep

**DOI:** 10.3390/ijms26051961

**Published:** 2025-02-24

**Authors:** Siyu Ren, Yuan Liu, Yajing Guo, Zhihui Zhao, Jingjing Cui, Mingna Li, Jiqing Wang

**Affiliations:** Key Laboratory of Herbivorous Animal Biotechnology, Faculty of Animal Science and Technology, Gansu Agricultural University, Lanzhou 730070, China; rsy4523@163.com (S.R.); ly482098@163.com (Y.L.); gyjgsau@163.com (Y.G.); 15934303408@163.com (Z.Z.); cuijj79@163.com (J.C.)

**Keywords:** TGF-β1, novel-m0297-5p, *WNT5A*, small-tailed Han sheep, follicles, granular cell

## Abstract

MiRNAs regulate follicle development and atresia, steroid production, granulosa cell (GC) proliferation, and apoptosis. However, the target genes and the functioning of novel miRNAs remain unexplored. We reveal the targeting relationship between novel-m0297-5p and *WNT5A* and the specific regulatory mechanism of GC proliferation in small-tailed Han sheep using whole transcriptomic sequencing. We performed whole transcriptomic sequencing on small-tailed Han sheep ovarian GCs supplemented with 10 ng/mL of transforming growth factor-β1 (TGF-β1) during the early stages. This led to identifying the differential expression of novel-m0297-5p and Wnt family member 5A (*WNT5A*) and predicting their targeting relationship. Based on this, we hypothesized that TGF-β1 could mediate novel-m0297-5p targeting *WNT5A* to participate in the proliferation process of GCs in small-tailed sheep. We confirmed the relationship between TGF-β1 and both novel-m0297-5p and *WNT5A*. The mimicry of novel-m0297-5p inhibited GC activity and proliferation. However, the inhibition of novel-m0297-5p yielded the opposite effect. We validated the binding site for novel m0297-5p within the 3′UTR of *WNT5A* using dual-luciferase reporter gene. TGF-β1 alleviated the impact induced by the mimicry of novel-m0297-5p on cell viability. Inhibitor co-transfection for both novel-m0297-5p and si-WNT5A suppressed the granulocyte proliferation induced by novel-m0297-5p inhibition. These findings suggest that TGF-β1 can mediate the inhibitory effect of novel-m0297-5p targeting *WNT5A* on GC proliferation and activity in small-tailed Han sheep. This study provides an experimental basis for research on the biological function of GCs and their impact on follicle development.

## 1. Introduction

Small-tailed Han sheep are one of the sheep breeds with high fertility in China. This breed has excellent growth and development, stable genetic performance, excellent reproductive performance, perennial estrus, and other excellent characteristics [1]. The ovary is the main reproductive organ of a female animal, and the follicle is the basic unit of the ovary. Granulosa cells (GCs), a key cell group within the follicles, play a crucial role in follicle development through their proliferation, apoptosis, and differentiation [2]. GCs provide protective and nutritional support for oocytes while secreting the hormones (e.g., estrogen) and growth factors [e.g., transforming growth factor (TGF), insulin-like growth factor (IGF), etc.] that interact with oocytes to regulate follicle maturation, ovulation, and fertilization [3,4,5,6,7]. Therefore, an in-depth investigation of the regulatory mechanisms of GC proliferation and apoptosis creates an opportunity to improve the fertility of female animals.

MiRNAs are critical in regulating follicle development and atresia, steroid production, proliferation, and the apoptosis of cumulus cells and GCs [8]. Studies have found that miR-133b stimulates ovarian estradiol synthesis by targeting *Foxl2* [9]. miR-27a-3p inhibits the proliferation of mouse granular cells (mGCs) by inhibiting the expression of the target genes *Vangl1* and *Vangl2* [10]. miR-223 inhibits the proliferation of chicken ovarian granule cells and the synthesis of steroid hormones by targeting and binding *CRIM1* [11]. miR-10b inhibits the proliferation of goat ovarian GCs by targeting the brain-derived neurotrophic factor (*BDNF*) [12]. miR-27a-3p can inhibit *CYP19A1* expression and 17-β estradiol (E2) synthesis in Hu sheep GCs [13]. miR-134-3p controls the proliferation and apoptosis of GCs in sheep by influencing the TGF-β/PI3K/AKT pathway [14]. miR-125b can inhibit GCs proliferation and steroidogenesis in sheep [15]. All of the above studies indicate that miRNAs can participate in the growth and proliferation of mammalian GCs and affect ovulation rates and litter size. However, more novel miRNAs are expected to be further explored and identified, and their important biological functions and regulatory mechanisms will be revealed.

Transforming growth factor-β1 (TGF-β1) is a multifunctional growth factor that is crucial in regulating various physiological processes, including embryonic growth and development [16], tissue fibrosis [17], immune cell development and differentiation [18], cell proliferation and apoptosis [19], and various pathologies. Frantz et al. found that TGF-β1 is highly expressed in various cell types (e.g., epithelial cells, GCs, and oocytes) and ovarian tissues [20]. This expression allows TGF-β1 to participate in ovulation, oocyte development, follicle development, and signal transmission between GCs and other functions [21]. Studies have found that TGF-β1 is one of the key regulatory factors of ovum origin, which plays a role in physiological processes such as ovarian GC proliferation and oocyte maturation, and can regulate several key GC enzymes, which play an important role in maintaining an ideal ovarian environment [22]. Yao et al. found, in a study of the GCs of Hu sheep, that lncRNA FDNCR sponged adsorbed miR-543-3p, preventing miR-543-3p from binding to *DCN* 3′UTR; activated the reverse transcription of Hu sheep *DCN*; inhibited the TGF-β pathway; decreased the expression of *TGF-β1*; and promoted the apoptosis of GCs [23]. Zhou et al. found in pig GCs that let-7g can induce GC apoptosis by targeting TGF-β1 [24]. Previously, we demonstrated that TGF-β1 exerts significant biological effects on the ovarian GCs of small-tailed Han sheep. Specifically, TGF-β1 treatment markedly enhanced cell viability and proliferation rates while suppressing apoptosis [25]. Subsequently, full transcriptome sequencing was performed on these supplemented small-tailed Han sheep ovarian GCs treated with 10 ng/mL of TGF-β1 to identify the differential expression patterns of novel-m0297-5p and *WNT5A*, along with their predicted targeting relationship. However, the biological functions of novel-m0297-5p and *WNT5A* in ovarian GCs from small-tailed Han sheep remain unclear.

The *WNT5A* gene is an important ligand in the Wnt family, a key molecule that regulates multiple signal networks. *WNT5A* plays a key regulatory role in stem cell differentiation and germ cell genesis [26]. Studies have shown that the *WNT5A* gene in mice is differentially expressed during follicle development, and inhibiting steroid production in atretic follicles affects follicle development [27]. Specifically, knocking out *WNT5A* in mouse ovarian GCs can promote follicular luteinization and reduce the ovulation rate [27]. Other studies have confirmed that *WNT5A* is a key regulator of follicle development and gonadotropin signaling [28]. The above studies indicate that *WNT5A* plays an important role in the development of follicles, but its effect on the GCs of small-tailed Han sheep has not been reported.

Based on the above research background, we hypothesized that TGF-β1 could mediate novel-m0297-5p targeting *WNT5A* to participate in the proliferation process of small-tailed Han sheep GCs, but this needed to be further verified by a large number of experiments. This study investigated the biological function of novel-m0297-5p in GCs by inhibiting and overexpressing novel-m0297-5p in cultured small-tailed Han sheep ovarian GCs. At the same time, the targeting relationship between the novel-m0297-5p and *WNT5A* was verified. Then, 10 ng/mL of TGF-β1-treated cells were used as the model to demonstrate whether TGF-β1 could mediate the regulatory mechanism of novel-m0297-5p targeting *WNT5A*, which is involved in the proliferation of small-tailed Han sheep GCs. This study provides a theoretical and experimental basis for further research on the potential functions of novel-m0297-5p and *WNT5A* in GCs and provides guidance for improving the productivity of small-tailed Han sheep.

## 2. Results

### 2.1. Novel-m0297-5p Secondary Structure and Target Gene Binding Site Prediction

**Figure 1:**
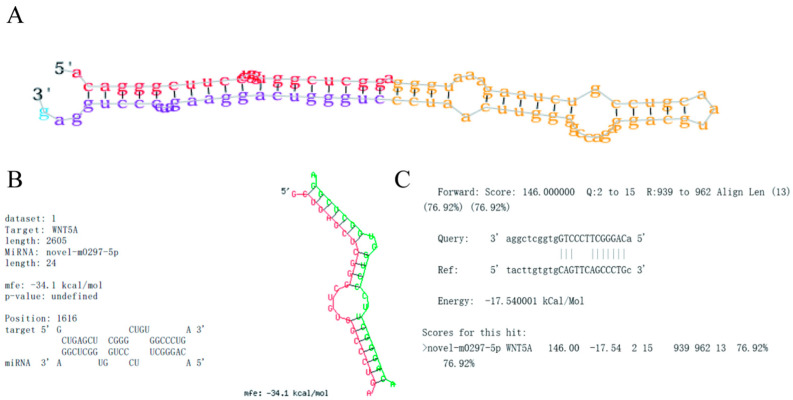
Novel-m0297-5p secondary structure and *WNT5A* binding site prediction. (**A**) Novel-m0297-5p secondary structure prediction; (**B**,**C**) represent the prediction of the binding sites of novel-m0297-5p and *WNT5A*. (**B**) miRanda determines the binding sites of novel-m0297-5p and *WNT5A*; and (**C**) RNAhybrid determines the binding sites of novel-m0297-5p and *WNT5A*. Note: (**A**) red: mature sequence; yellow: annular structure; and purple: start sequence. (**B**) red: WNT5A sequence; green: novel-m0297-5p sequence.

The secondary structure of the novel-m0297-5p precursor was consistent with the secondary structure characteristics determined using mirDeep2. The secondary structure of the novel-m0297-5p precursor had a typical stem ring structure (Figure 1A). The mature sequence of novel-m0297-5p was 5′-AcagGGCttccCTGGtGGCTCGGA-3′.

According to the complementary matching principle between target genes and miRNAs, the online software miRanda “http://www.bioinformatics.com.cn (accessed on 20 April 2024)”and RNAhybrid “http://www.bioinformatics.com.cn (accessed on 20 April 2024)” were used to predict the binding sites of the *WNT5A* gene 3′UTR and novel-m0297-5p, and a seed binding site, CAGGGCT, was found between them (Figure 1B,C).

### 2.2. Effects of Novel-m0297-5p on GC Proliferation

**Figure 2:**
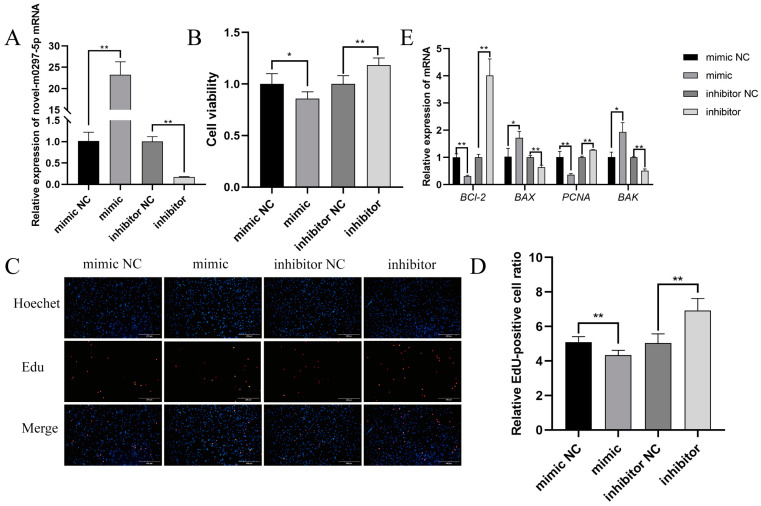
Effects of novel-m0297-5p on the proliferation and apoptosis of GCs. (**A**) shows the relative expression of novel-m0297-5p in the ovarian GCs of small-tailed Han sheep detected by the RT-qPCR. (**B**) is the statistical result of cell viability detected by the CCK-8. (**C**) is the result of cell proliferation detected by the EdU. The EdU (red) fluorescent label is cell proliferation, and the DAPI (blue) fluorescent label is the nucleus. Scale bar, 200 µm. (**D**) is the statistical result of the EdU-stained cells. (**E**) is the expression level of the genes related to proliferation and apoptosis. Note: * means a significant difference (*p* < 0.05); and ** means an extremely significant difference (*p* < 0.01) (see below as well).

To investigate the effect of novel-m0297-5p on the proliferation of small-tailed Han sheep GCs, the mimic, inhibitor, and negative control (a mimic NC and inhibitor NC) novel-m0297-5p were transfected into the ovarian GCs of small-tailed Han sheep. The effectiveness of the novel-m0297-5p mimics and inhibitors was validated using an RT-qPCR. The expression of novel-m0297-5p after transfection with the mimic was significantly upregulated compared with the mimic NC (*p* < 0.01; Figure 2A). The expression of novel-m0297-5p was significantly downregulated after transfection with the inhibitor (*p* < 0.01), indicating that the novel-m0297-5p mimic and inhibitor had been transfected successfully.

The CCK-8 method was used to detect the ovarian GC viability of small-tailed Han sheep. The novel-m0297-5p mimic significantly inhibited cell viability (*p* < 0.05), while the inhibitor significantly increased cell viability (*p* < 0.01) (Figure 2B).

An EdU test was conducted to further investigate the effect of novel-m0297-5p on cell proliferation. The mimic significantly decreased the EdU-positive rate of the cells (*p* < 0.01; Figure 2C), while the inhibitor significantly increased the EdU-positive rate of the cells (*p* < 0.01) (Figure 2D). The results indicated that novel-m0297-5p could inhibit the proliferation of ovarian GCs in small-tailed Han sheep.

The novel-m0297-5p mimic significantly decreased the relative mRNA expressions of *PCNA* and *Bcl-2* (*p* < 0.01; Figure 2E) and significantly increased the relative mRNA expressions of *Bax* and *BAK* (*p* < 0.05; Figure 2E). However, the inhibitor NC showed the opposite result. This result was consistent with the CCK-8 and EdU results, confirming the accuracy of the CCK-8 and EdU results.

### 2.3. Targeting Validation Between Novel-m0297-5p and WNT5A

**Figure 3:**
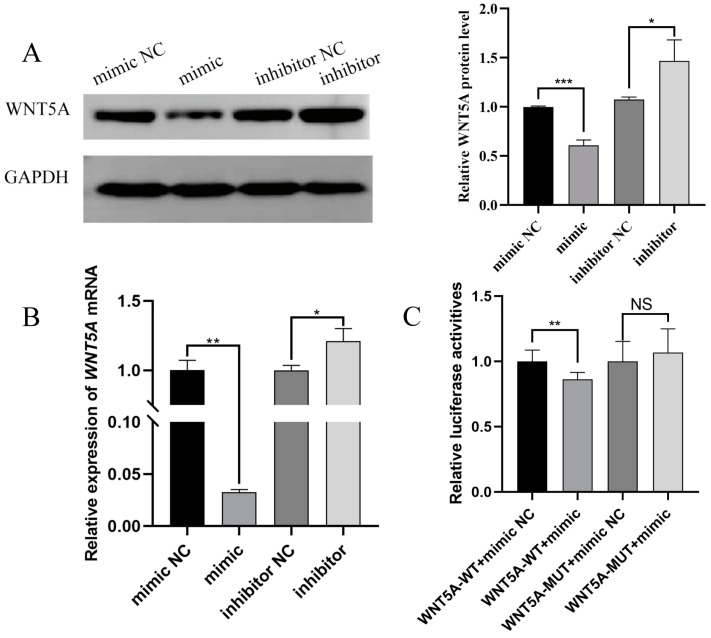
Target relationship between novel-m0297-5p and *WNT5A*. A and B are the relative expression levels of the *WNT5A* gene mRNA and its coding protein in ovarian GCs. (**A**) is the result of the Western blot and gray value analyses. (**B**) is the result of the qRT-PCR. (**C**) demonstrates the relative luciferase activity of WNT5A-WT and MUT after transfection with a novel-m0297-5p mimic or mimic NC. Note: * means a significant difference (*p* < 0.05); ** means an extremely significant difference (*p* < 0.01). *** means a highly significant difference (*p* < 0.001). and NS means not significant (*p* ≥ 0.05).

The novel-m0297-5p mimic, inhibitor, and negative control (NC) were transfected into GCs to investigate the regulatory relationship between novel-m0297-5p and *WNT5A*. RT-qPCR and Western blot analyses were used to detect the relative expression of the *WNT5A* gene and protein. The results of the Western blot are shown in Figure 3A. The novel-m0297-5p mimic significantly reduced the relative expression of the WNT5A protein (*p* < 0.01). However, the relative expression of the WNT5A protein was significantly increased with the inhibitor (*p* > 0.05). The RT-qPCR results are shown in Figure 3B. The novel m0297-5p mimic significantly decreased the relative expression level of *WNT5A* mRNA (*p* < 0.01), while the inhibitor significantly increased the relative expression level of *WNT5A* mRNA (*p* > 0.05), which was consistent with its protein expression trend.

A dual-luciferase assay explored the targeting relationship between novel-m0297-5p and *WNT5A* to further verify the direct regulatory mechanism between novel-m0297-5p and *WNT5A*. The novel-m0297-5p mimic significantly reduced the relative fluorescence activity of the *WNT5A* wild-type dual-luciferase carrier (*p* < 0.001; Figure 3C), while it did not affect the relative fluorescence activity of the *WNT5A* binding site mutant dual-luciferase carrier (*p* ≥ 0.05; Figure 3C). Novel-m0297-5p can bind to the 3′UTR region of the *WNT5A* gene to target *WNT5A*.

### 2.4. TGF-β1 Mediates Novel-m0297-5p Targeting WNT5A to Participate in Cell Proliferation

We compared the homology of TGF-β1 proprotein preproprotein (Human, NP_000651.3) and TGF-β1 proprotein preproprotein (Sheep, NP_001009400) and found that the homology reached 94.28% (Figure S1). Therefore, we selected the recombinant human TGF-β1 protein for the follow-up experiments.

**Figure 4:**
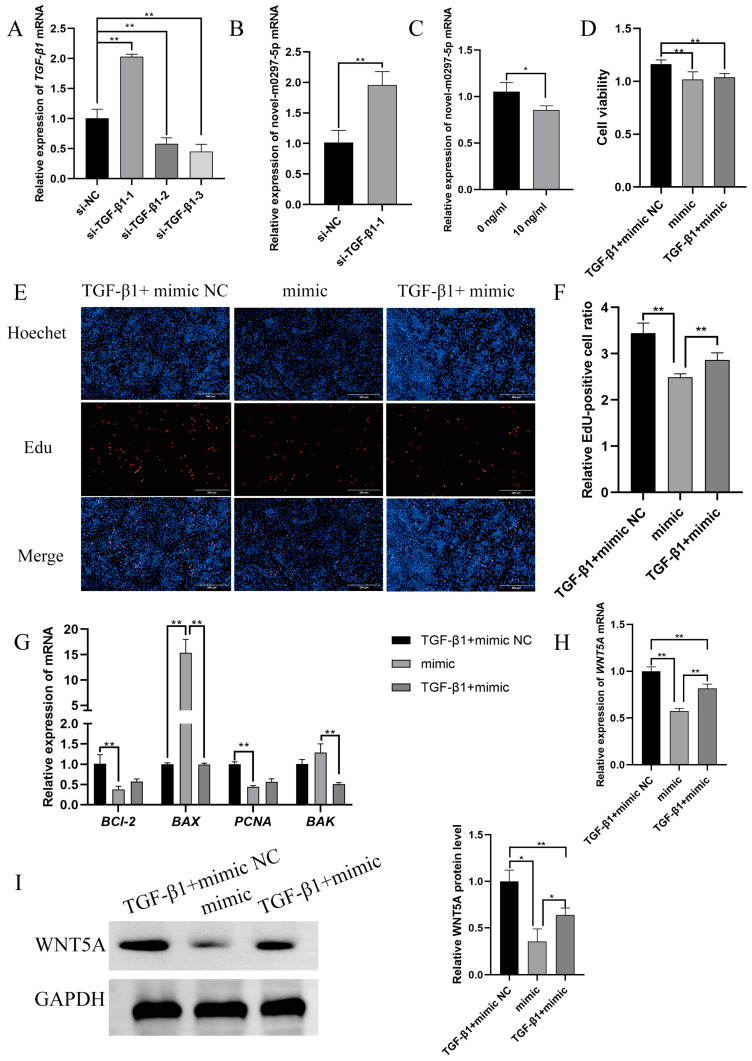
Effects of TGF-β1 addition and novel-m0297-5p transfection on GC viability, proliferation, and apoptosis. (**A**) is the siRNA screening of TGF-β1. (**B**) is the relative expression of novel-m0297-5p in GCs after TGF-β1 knockdown. (**C**) is the relative expression of novel-m0297-5p in 10 ng/mL of TGF-β1 GCs. (**D**) is the statistical result of the cell viability detected by the CCK-8. (**E**,**F**) are the EdU results of cell proliferation. Scale bar, 200 µm. (**G**) is the expression level of the genes related to proliferation and apoptosis. (**H**,**I**) are the relative expression levels of the *WNT5A* gene mRNA and its coding protein in ovarian GCs. (**H**) is the result of the qRT-PCR. (**I**) is the result of the Western blot and gray value analyses. Note: * means a significant difference (*p* < 0.05), and ** means an extremely significant difference (*p* < 0.01).

We designed three siRNA sequences to knock down the expression level of *TGF-β1* to verify the regulatory relationship between TGF-β1 and novel-m0297-5p. As can be seen from Figure 4A, the si-TGF-β1-3 knockdown efficiency was the best (*p* < 0.01). Therefore, si-TGF-β1-3 was selected for the subsequent tests. Subsequently, an RT-qPCR was used to detect the relative expression of novel-m0297-5p in GCs after TGF-β1 knockdown. The expression level of novel-m0297-5p significantly increased after TGF-β1 knockdown (*p* < 0.01; Figure 4B) compared with the control group. Then, the relative expression of novel-m0297-5p in GCs after adding 10 ng/mL of TGF-β1 was detected. The expression level of novel-m0297-5p in the 10 ng/mL TGF-β1 group significantly decreased (*p* < 0.05; Figure 4C) compared with the control group.

TGF-β1 and a novel-m0297-5p mimic were added to the ovarian GCs of small-tailed Han sheep using a rescue test to further verify whether TGF-β1 could mediate the involvement of novel-m0297-5p in cell proliferation. The CCK-8, EdU, and qRT-PCR methods were used to detect ovarian GC viability, the cell proliferation rate, and the relative expression of the genes related to proliferation and apoptosis in small-tailed Han sheep. Compared with the mimic group, the addition of TGF-β1 did alleviate the decrease in cell viability caused by the mimic, but the difference was insignificant (*p* = 0.79; Figure 4D).

The EdU staining results are shown in Figure 4E,F. Compared with the mimic group, adding TGF-β1 could significantly increase the EdU-positive rate of the GCs transfected with the novel m0297-5p mimic (*p* < 0.01).

The results of the qRT-PCR are shown in Figure 4G. The relative expression levels of the *PCNA* (*p* = 0.10) and *Bcl-2* (*p* = 0.27) mRNA in the TGF-β1 group were increased, but the differences were insignificant compared with the mimic group. The relative mRNA expressions of *Bax* and *BAK* significantly decreased (*p* < 0.01), in agreement with the CCK-8 and EdU results and further confirming the accuracy of the CCK-8 and EdU results.

TGF-β1 and the novel-m0297-5p mimic were added to the ovarian GCs of small-tailed Han sheep using a rescue test to verify whether TGF-β1 could mediate novel-m0297-5p targeting the *WNT5A* involved in cell proliferation. The RT-qPCR and Western blot were used to detect the relative expression of the *WNT5A* gene and protein. The results of the RT-qPCR are shown in Figure 4H. Compared with the mimic group, the addition of TGF-β1 significantly increased the relative expression of the *WNT5A* mRNA (*p* < 0.01). The results of the Western blot are shown in Figure 4I. Compared with the mimic group, the addition of TGF-β1 could significantly increase the relative expression of the WNT5A protein (*p* > 0.05), which was consistent with the trend of mRNA expression. These results indicated that TGF-β1 could mediate novel-m0297-5p targeting *WNT5A* to participate in GC proliferation.

### 2.5. Novel-m0297-5p Targeting WNT5A to Participate in Cell Proliferation

**Figure 5:**
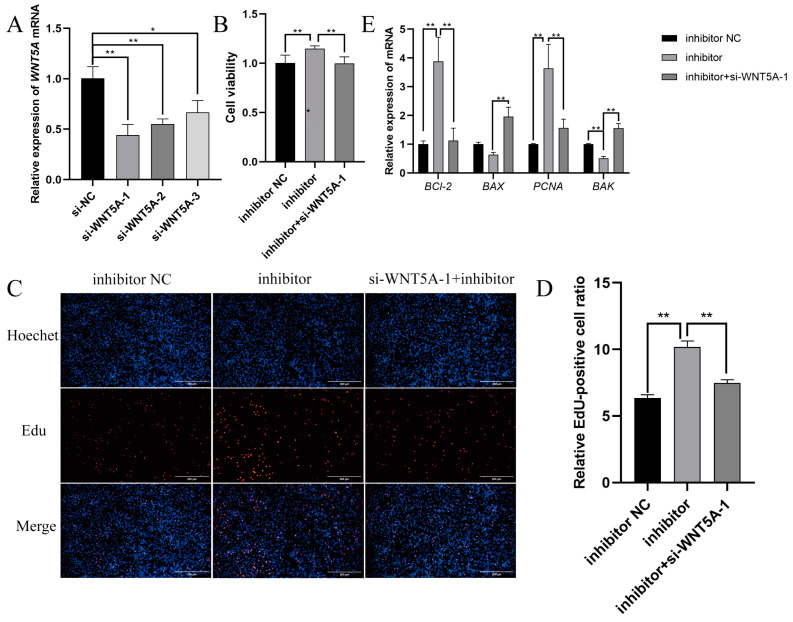
Effects of novel-m0297-5p inhibitor co-transfection with si-WNT5A-1 on GC viability, proliferation, and apoptosis. (**A**) is the siRNA screening of *WNT5A*. (**B**) is the statistical result of cell viability detected by the CCK-8. (**C**,**D**) are the results of cell proliferation detected by the EdU. Scale bar, 200 µm. (**E**) is the expression level of the genes related to proliferation and apoptosis. Note: * means a significant difference (*p* < 0.05); and ** means an extremely significant difference (*p* < 0.01).

We designed three siRNA sequences to knock down the expression of *WNT5A* to further verify whether TGF-β1 can mediate novel-m0297-5p targeting *WNT5A* to participate in cell proliferation. si-WNT5A-1 had the best knockdown efficiency. Therefore, si-WNT5A-1 was selected for a follow-up test (*p* < 0.01) (Figure 5A). Subsequently, the si-WNT5A-1 and novel-m0297-5p inhibitors were simultaneously transfected into small-tailed Han sheep ovarian GCs by a rescue assay. The CCK-8, EdU, and qRT-PCR methods were used to detect the viability, cell proliferation rate, and relative expression levels of the genes related to the proliferation and apoptosis of small-tailed Han sheep ovarian GCs.

The results of the CCK-8 are shown in Figure 5B. The addition of si-WNT5A-1 significantly reduced the increase of GC viability induced by the inhibitor compared with the novel-m0297-5p inhibitor group (*p* < 0.01). The EdU results are shown in Figure 5C,D. The addition of si-WNT5A-1 significantly reduced the increase in the EdU-positive rate of the cells induced by an inhibitor compared with the novel-m0297-5p inhibitor group (*p* < 0.01). The RT-qPCR results are shown in Figure 5E. The *PCNA* and *Bcl-2* mRNA expression levels were significantly decreased in the novel m0297-5p inhibitor group co-transfected with the si-WNT5A-1 inhibitor group compared with the novel m0297-5p inhibitor group (*p* < 0.01). The mRNA expressions of *Bax* and *BAK* were significantly increased (*p* < 0.01). This result is consistent with the CCK-8 and EdU results, further confirming the accuracy of the CCK-8 and EdU results.

## 3. Discussion

Studies have demonstrated that the apoptosis of ovarian GCs in females can lead to follicular atresia [29], reducing ovulation rates and subsequently impacting reproductive performance. GCs are the microenvironmental regulators of follicle growth and oocyte maturation, and their proliferation and apoptosis play key roles in follicle development. When GC proliferation ability decreases, the apoptosis rate increases and hormone secretion is disturbed, follicular development becoming abnormal [2]. The proliferation and apoptosis of GCs play a pivotal role in follicular development. Therefore, it is crucial to fully elucidate the regulatory mechanisms governing GC proliferation and apoptosis during this process. Increasingly, studies have focused on the involvement of miRNAs in regulating GC proliferation and apoptosis. However, most of these studies primarily concentrated on targeted gene mining and the functional analysis of known miRNAs, with limited investigation into novel mRNA. This study used small-tailed Han sheep ovarian GCs supplemented with 10 ng/mL of TGF-β1 to screen the differential expression of novel-m0297-5p and *WNT5A* through a small RNA-Seq. Additionally, the predicted targeting relationship between *WNT5A* and novel-m0297-5p was examined. We used CCK8 and EdU incorporation assays, Western blotting analysis, dual-luciferase reporter assays, RT-qPCR experiments, and overexpression and silencing techniques. We found that TGF-β1 can mediate the targeting effect of novel m0297-5p on *WNT5A*, leading to its participation in the proliferation process of ovarian GCs from small-tailed Han sheep.

In this study, we identified TGF-β1 as a crucial regulator of GC viability and proliferation, playing a significant role in the regulatory processes of follicle recruitment, development, and ovulation [30,31,32]. It has been found that miRNA can regulate GC function and follicle development by targeting the TGF-β signaling pathway [33,34,35]. miR-424 promotes the proliferation of bovine GCs by targeting *SMAD7* [36]. miR-181a and miR-1343 affect the proliferation and apoptosis of porcine GCs by targeting TGF-β1 [37,38]. Li et al. [39] demonstrated that adding 10 ng/mL of TGF-β1 effectively inhibits apoptosis and promotes proliferation in porcine ovarian GCs. Similarly, Yin et al. [40] found that 10 ng/mL of TGF-β1 significantly enhances *GDNF* and *Furin* expression, thereby promoting viability and proliferation in human luteal GCs. These findings suggest that the supplementation of TGF-β1 improves viability and proliferation while inhibiting apoptosis in ovarian GCs [25]. However, the precise mechanism by which TGF-β1 regulates GC proliferation remains unclear, particularly regarding the involvement of TGF-β1-mediated miRNA regulation. Therefore, our study aimed to validate the sequencing data by demonstrating that adding 10 ng/mL of TGF-β1 significantly reduces novel-m0297-5p mRNA expression levels. Conversely, the knockdown of *TGF-β1* markedly increases novel-m0297-5p mRNA expression levels, further confirming the reliability of our sequencing results. Nevertheless, despite these findings shedding light on the potential mechanisms involved in ovarian GC function modulation by novel-m0297-5p miRNA regulation, its specific biological functions remain elusive, requiring further experimental investigations.

Subsequently, we investigated the biological function of novel-m0297-5p. The results from the CCK-8 and EdU assays demonstrated that the mimic of novel-m0297-5p effectively suppressed cell proliferation, while the inhibitor yielded the opposite effect. Furthermore, the alterations in the mRNA expression levels of the proliferation marker genes (*PCNA*) and apoptosis marker genes (*Bcl-2*, *Bax*, *BAK*) were consistent with these findings. These results strongly suggest that novel-m0297-5p actively regulates cell proliferation and is an essential modulator of granular cell activity in small-tailed Han sheep. With advancing research, increasing attention has been devoted to elucidating the role of non-coding RNA in GC proliferation. For instance, miR-26b downregulates *SMAD1* expression, inhibiting proliferation and promoting apoptosis in yak GCs [41]. Additionally, miR-34a promotes apoptosis in porcine GCs by targeting the *INHBB* gene [42]. Moreover, novel miRNA Y-56 inhibits the proliferation and cell cycle progression of porcine skeletal muscle satellite cells (PSCs) through *IGF-1R* targeting [43]. miR-216a-3p can inhibit the proliferation and promote the apoptosis of GCs [44]. LNCRNA-412.25 regulates GC proliferation and apoptosis in Hu sheep by binding to miR-346 and then activating the LIF/STAT3 pathway [45]. oar-miR-150 promotes the apoptosis of sheep ovarian GCs by targeting the *STAR* gene [46]. Novel 58-5p inhibits steroid hormone secretion by targeting *SREBF1* and promotes the apoptosis of ovarian GCs under high concentrations of prolactin (PRL) [47]. In summary, although much remains to be explored regarding their functions, especially concerning mining and identifying novel miRNAs, these findings provide valuable experimental evidence for future investigations into the involvement of non-coding RNAs in cellular processes.

More studies have consistently reported that miRNA can bind to the 3′ untranslated region (UTR) of mRNA, inhibiting gene translation or directly promoting mRNA degradation to regulate gene expression. Subsequently, we employed bioinformatics software RNAhybrid (Version 2.1.2) “https://bibiserv.cebitec.uni-bielefeld.de (accessed on 20 April 2024)” and miRanda “http://www.bioinformatics.com.cn (accessed on 20 April 2024)” for the prediction and identified novel-m0297-5p as a potential target for *WNT5A*. The dual-luciferase reporter gene assay confirmed the specific targeting of *WNT5A* by novel-m0297-5p, significantly suppressing its expression at both the transcriptional and translational levels. These findings unequivocally establish *WNT5A* as a pivotal target gene for novel-m0297-5p. Although this study did not extensively investigate the biological function of *WNT5A*, the literature review suggests its crucial involvement in the ovarian ovulation and cell proliferation processes. The Wnt signaling pathway can affect follicle growth by regulating estrogen synthesis, oocyte development, and GC apoptosis [48,49,50]. As a Wnt protein, Wnt5A regulates the proliferation and apoptosis of GCs and has been recognized as a regulator of follicular development and gonadotropin reactivity in adult ovaries [51]. Abedini et al. found that the GC-specific inactivation of Wnt5a results in female subfertility associated with increased follicular atresia and decreased rates of ovulation [28]. Studies have demonstrated an increase in the ovarian GC expression of *WNT5A* during mouse ovulation, while also revealing that it enhances the expression of the cumulus-expanding gene *HAS2* through the JNK and AKT signaling pathways in human granulosa-like KGN cells [52]. Sun et al. demonstrated that scutellarin produced anti-apoptotic effects on Zearalenone (ZEA, F-2 toxin)-induced mouse ovarian GCs by targeting *WNT5A* [53]. Furthermore, in the other literature, it has been indicated that miR-26a-5p negatively regulates tumor proliferation and invasion while promoting cell apoptosis by targeting the expression of the *WNT5A* gene [54]. These reports indirectly support the involvement of *WNT5A* in cellular apoptosis and ovarian activity among female animals, thereby providing valuable insights for this study.

The role and regulatory relationship of TGF-β1, novel-m0297-5p, and *WNT5A* in the ovarian GCs of small-tailed Han sheep have now been elucidated. Furthermore, the negative regulatory association between TGF-β1 and novel-m0297-5p and the targeting correlation between novel-m0297-5p and *WNT5A* were confirmed. Consequently, we venture to speculate that TGF-β1 may mediate the targeting of novel-m0297-5p toward *WNT5A* to participate in the proliferation of GCs in small-tailed sheep. This hypothesis has been validated through two rescue experiments. Thus, this study unveils a new pathway by which TGF-β1 regulates the proliferation of ovarian GCs in small-tailed Han sheep—namely, a fresh mechanism involving TGF-β1-novel-m0297-5p-*WNT5A* that impacts GC proliferation in these animals. Despite making significant progress in our research, certain limitations remain. Primarily relying on in vitro cell culture models for our experiments necessitates further validation using in vivo models for more robust results. Additionally, although we have initially uncovered a new regulatory network comprising TGF-β1-novel-m0297-5p-*WNT5A*, additional analyses are required to elucidate the specific signaling pathways and molecular interactions involved in GC proliferation.

## 4. Materials and Methods

All animal procedures in this study were approved by the Animal Experiment Ethics Committee of Gansu Agricultural University (approval number GSAU-ETH-AST-2021-028).

### 4.1. Sample Collection

One hundred ewes, with normal growth and development, of 2–3-year-old small-tailed Han sheep were collected from the Kangtai slaughterhouse, Linxia City. After removing the excess tissue around them and sterilizing with 75% alcohol, the ovary were placed in normal saline containing a double antibody (100 IU/mL of penicillin and 100 μg/mL of streptomycin) preheated to 37 °C and transported back to the cell culture room within 2 h.

### 4.2. Culture, Passage, and Identification of Ovarian GCs

The surface of the ovary was cleaned with 75% alcohol, rinsed three times with sterile 0.9% NaCl solution, and then brought into the sterile laboratory with normal saline containing 1% diamante. Follicular fluid with a diameter of more than 4 mm and no collapsed follicles was extracted with a 1 mL disposable sterile syringe, filtered with a 400-mesh cell filter, centrifuged at 1000 rpm, and centrifuged at room temperature for 5 min. DMEM/F12 medium (Gibco, Grand Ialand, NY, USA) suspension centrifugation was repeated twice.The cells were maintained in complete DMEM/F12 medium (containing: DMEM/F12 basal medium (Gibco, Grand Ialand, NY, USA), 10% fetal bovine serum (FBS; Gibco, Grand Ialand, NY, USA) and 1% triple antibody (penicillin-streptomycin-amphotericin B)) at 37 °C under 5% CO_2_. The fluid was changed every 48 h.

The passage can be conducted when 80–90% of the cells are confluent. The cells were washed twice with PBS and digested with 0.25% trypsin (Gibco, Grand Ialand, NY, USA) (37 °C, 3–5 min). When most of the cells fell from the bottom of the bottle, the digestion was terminated by adding a complete medium of equal volume and centrifugation at 1000 rpm for 5 min at room temperature. Finally, the sample was re-suspended with a complete medium, inoculated in a cell culture bottle, and cultured in a cell culture box.

### 4.3. TGF-β1 Treatment and Cell Transfection

Based on the TGF-β1 protein sequences published on the NCBI website, BLAST “https://blast.ncbi.nlm.nih.gov/Blast.cgi (accessed on 8 May 2024)” was used for the homology analysis. Homology comparisons of the amino acid sequences were performed using DNAMAN 8.0 (Lynnon Biosoft, San Ramon, CA, USA).

The GCs were inoculated in 24-well plates for the RNA sample extraction and EdU assay or 96-well plates for the CCK8 assay. Human TGF-β1 recombinant protein (Bioss, Beijing, China) was added when the cell confluence reached 50–60%, and the final concentration was 10 ng/mL.

The siRNAs were designed and synthesized by Hongxun Biotechnology Co., Ltd. (Suzhou, China). The siRNA sequence is shown in Table 1. Three siRNAs were designed for each gene, and the best siRNA was selected for the subsequent experiments.

The cell culture was performed using fresh media before the RNA transfection using the Invigentech INVI DNA RNA transfection reagent (Invigentech, Carlsbad, CA, USA), transfected with novel-m0297-5p mimic, novel-m0297-5p mimic NC, novel-m0297-5p inhibitor, novel-m0297-5p inhibitor siRNA of NC, TGF-β1, and *WNT5A*, according to the manufacturer’s instructions. The sequences of the novel-m0297-5p mimic and inhibitor and their negative control (NC) are shown in Table 2. The cells were harvested for RNA extraction 48 h after transfection. The mimic, inhibitor, and NC of novel-m0297-5p were synthesized by Servicebio Biotechnology Co., Ltd. (Wuhan, China). The sequence of novel-m0297-5p is as follows: 5′-ACAGGGCTTCCCTGGTGGCTCGGA-3′.

### 4.4. Dual-Luciferase Assay

The differential expression of novel-m0297-5p and *WNT5A* was screened, and *WNT5A* was predicted to be the target gene of novel-m0297-5p through the interaction network, based on the transcriptome sequencing data of a previous research group. RNAhybrid “https://bibiserv.cebitec.uni-bielefeld.de (accessed on 20 April 2024)” and miRanda “http://www.bioinformatics.com.cn (accessed on 20 April 2024)” were used to predict *WNT5A* and the binding site of the mRNA, 3′UTR, to novel-m0297-5p. The pmiRGLO-WNT5A-WT and pmiRGLO-WNT5A-MUT were synthesized by Jin Weizhi Company (Suzhou, China). The luciferase reporter plasmid (wild-type or mutant pmiRGLO-WNT5A) was transfected into HEK293T ((Gibco, Grand Ialand, NY, USA) cells with a novel-m0297-5p mimic or novel-m0297-5p mimic NC, respectively. It was 48 h after transfection that the cells were analyzed with the Dual-Luciferase^®^ Reporter Assay System kit (Promega, Madison, WI, USA). The activity of sea kidney luciferase (R-luc) was standardized using firefly luciferase (F-luc) to assess the reporter gene translation efficiency, with six replicates per trial.

### 4.5. Total RNA Extraction, Reverse Transcription, and Real-Time Fluorescence Quantitative PCR (qRT-PCR) Test

The total RNA was extracted from the cells according to the instructions of the AG RNAex Pro RNA extraction reagent (Aikerui, Hunan, China). Then, the HiScript^®^II Q RT SuperMix for qPCR (+gDNA wiper) kit (Vazyme, GDNA Wiper) and the miRNA cDNA first chain synthesis kit (Aikerui, Hunan, China) were used to reverse-transcribe the mRNA and miRNA into cDNA, respectively. According to the instructions of the SYBR Green Pro Taq HS premixed qPCR kit (Aikerui, Hunan, China), the qRT-PCR analysis was performed. The 20 μL reaction system consisted of 2× SYBR^®^Green Pro Taq HS Premix, 10.0 μL; 0.4 μL of upper and downstream primers (10 nM); 1.0 μL of cDNA; and 8.2 μL of RNase-free ddH2O. The qRT-PCR reaction was performed using the ABI QuantStudioTM6 Flex qPCR instrument. The reaction procedure was as follows: predenatured at 95 °C for 30 s, denatured at 95 °C for 5 s, annealed at 60 °C for 30 s, and extended at 72 °C for 30 s for 40 cycles. mirDeep2 [55] was used to predict the secondary structure of the novel-m0297-5p. Primer Premier 5.0 [56] was used to design the primers (Table 3). BLAST [57] was used for the specific analyses. The primers were sent to YangLing Tianrun Aoke Biotechnology Co., Ltd. (Shanxi, China) for synthesis. *U6* and *GAPDH* were used as the internal controls of novel-m0297-5p and mRNA. There were three replicates of novel-m0297-5p and mRNA in each group. The primers for novel-m0297-5p were synthesized as follows: forward primer 5′–AATTAACAGGGCTTCCCTGGTGG–3′ and reverse primer 5′–TCGCTCCACCAACTAAGAA–3′. The primers for *U6* were synthesized as follows: forward primer 5′–ACGGACAGGATTGACAGATT–3′ and reverse primer 5′–TCGCTCCACCAACTAAGAA–3′. A relative quantitative analysis was performed using the 2^−ΔΔCt^ method, and three replicates were set for each group.

### 4.6. Western Blot Test

The cells were cleaned with PBS, and the total protein was extracted from the Radio Immunoprecipitation Assay (RIPA) lysate buffer (including PMSF) (Servicebio, Wuhan, China). A BCA kit (Cowin Biotech Co., Ltd., Jiangsu, China) was used to detect the total protein concentration by adding 5 × protein loading buffer (Servicebio, Wuhan, China) in a ratio of 4:1 and boiling at 98 °C for 5 min, before an ice bath for 5 min. The procedure was repeated three times, after which the solution was stored in a −80 °C refrigerator.

For the electrophoresis, 30 μg of denatured proteins were loaded into each well, using *GAPDH* as the loading control. and electrophoresed on 12% SDS-PAGE gel (Sodium dodecyl sulfate-polyacrylamide gel). Subsequently, the proteins were transferred onto PVDF (Polyvinylidene fluoride) membranes. Then, 5% skim milk powder was used and enclosed at room temperature for 1.5 h. Then, the rabbit primary antibody *WNT5A* (Affinity, OH, USA, 1:1000) and *GAPDH* (Affinity, OH, USA, 1:5000) were incubated at 4 °C overnight and washed with tris-buffered saline with Tween 20 (TBST). Next, the sample was treated with goat anti-Rabbit IgG(Affinity, OH, USA, 1:6000) and incubated at room temperature for 1 h. The film was washed, reacted with substrate electrochemiluminescence (ECL) for 5 min, then exposed to a chemiluminescence instrument, before the images were acquired.

### 4.7. Cell Viability and Proliferation Detection

According to the instructions, a cell counting kit-8 (CCK-8) (Biosharp, Beijing, China) was used to detect GC viability. Specifically, the cells were inoculated into 96-well plates and treated differently according to the experimental requirements. Then, 10 μL of CCK-8 solution was added to each well and incubated at 37 °C for 4 h. The optical density (OD) values of each group were measured at 450 nm with a Varioskan LUX multifunctional enzyme marker (Thermo Scientific, Waltham, MA, USA), and the cell viability was calculated. Five replicates were set for each group.

The proliferation rate of GCs in small-tailed sheep was analyzed using an EdU-555 cell proliferation assay kit (Biyuntian, Shanghai, China). Specifically, the cells were inoculated into 24-well plates for the different transfection treatments, preheated with a 2 × EdU working solution (20 μM) at 37 °C, added to 24-well plates in equal volume, and allowed to incubate for 2 h. Next, the culture medium was discarded, and 1 mL of fixing solution (4% paraformaldehyde) was added for 15 min. Later, the fixing solution was removed, and 1 mL of washing solution (PBS containing 3% BSA) was added three times for 5 min/time. The washing solution was removed, and 1 mL of permeable solution (PBS containing 0.3% Triton X-100) was added for 15 min. Then, the permeable solution was removed, and 1 mL of washing solution was added twice for 5 min/time. For the click reaction solution, the necessary resources were 430 μL of click reaction buffer, 20 μL of CuSO_4_, 1 μL of Azide555, and 50 μL of click additive solution, with a total volume of 500 μL. The reaction solution was prepared according to this system. First, 100 μL of the click reaction solution was added and incubated away from the light for 30 min. After removing the click reaction solution, 1 mL of washing solution was added three times for 5 min/time. Hoechst 33,342, diluted with 100 μL of PBS (*v*/*v*, 1:1000), was added and incubated away from the light for 10 min. Then, 1 mL of washing solution was added 3 times for 5 min/time. The fluorescence was detected under a microscope. Three replicates were set up in each group, and the cells were counted using ImageJ 1.54g software (National Institutes of Health, Bethesda, Montgomery, MD, USA) [58]. The cell proliferation rate was calculated as follows: cell proliferation rate (%) = number of proliferating cells/total number of cells × 100.

### 4.8. Statistical Analysis

SPSS 20.0 (IBM Corporation, Armonk, NY, USA) was used to analyze the data. An independent sample *t*-test was used for the analyses involving two groups, and a one-way analysis of variance (ANOVA) was used for the analyses involving comparisons of three groups or more. The Western blot results were quantitatively analyzed using Image J 1.54g software (National Institutes of Health, Bethesda, Montgomery, MD, USA) [58] and GraphPad Prism8.0.2 [59]. The data are presented as the “mean ± standard deviation”, with *p* < 0.05 indicating a significant difference and *p* < 0.01 indicating a high level of significant difference.

## 5. Conclusions

In summary, this study demonstrated that novel-m0297-5p significantly suppresses the expression of *WNT5A* by targeting its 3′UTR region. The overexpression of novel-m0297-5p markedly inhibits the viability and proliferation of the ovarian GCs in small-tailed Han sheep, downregulates the expression of proliferation-related marker genes, and upregulates the expression of pro-apoptotic marker genes, further confirming the impact of novel-m0297-5p targeting WNT5A on GC phenotypes. Additionally, we revealed a novel regulatory pathway involving TGF-β1–novel-m0297-5p–WNT5A in modulating the proliferation of ovarian GCs, providing new insights into the molecular mechanisms underlying follicular development in small-tailed Han sheep. These findings offer a theoretical foundation for further exploration of the molecular mechanisms regulating ovarian follicular development in small-tailed Han sheep.

## Figures and Tables

**Table 1:** siRNA sequence information.

Genes	Primer Sequence (5′→3′)
si-TGF-β1-1	Sense: GCGUGCUAAUGGUGGAAUACGTTAntisense: CGUAUUCCACCAUUAGCACGCTT
si-TGF-β1-2	Sense: GAGCUGUACCAGAAAUAUAGCTTAntisense: GCUAUAUUUCUGGUACAGCUCTT
si-TGF-β1-3	Sense: CCUGUGACAGUAAGGAUAACATTAntisense: UGUUAUCCUUACUGUCACAGGTT
si-WNT5A-1	Sense: AGGUUGUAAUAGAAGCUAAUUTTAntisense: AAUUAGCUUCUAUUACAACCUTT
si-WNT5A-2	Sense: GCGGCGACAACAUCGACUACGTTAntisense: CGUAGUCGAUGUUGUCGCCGCTT
si-WNT5A-3	Sense: CCGACUACUGCGUGCGCAACGTTAntisense: CGUUGCGCACGCAGUAGUCGGTT
si-NC	Sense: UUCUCCGAACGUGUCACGUdTdTAntisense: ACGUGACACGUUCGGAGAAdTdT

**Table 2:** Sequence information of the novel-m0297-5p mimic, inhibitor, and negative control (NC).

Genes	Sequence Information (5′→3′)
mimic	ACAGGGCUUCCCUGGUGGCUCGGAUCCGAGCCACCAGGGAAGCCCUGU
mimic NC	UCACAACCUCCUAGAAAGAGUAGAUCUACUCUUUCUAGGAGGUUGUGA
inhibitor	UCCGAGCCACCAGGGAAGCCCUGU
inhibitor NC	UCUACUCUUUCUAGGAGGUUGUGA

**Table 3:** Primer sequence information.

Genes	GenBank ID	Primer Sequence (5′–3′)	Product Length/bp
*Bax*	XM_027978592.2	F:TCTCCCCGAGAGGTCTTTTT	177
		R:TCGAAGGAAGTCCAATGTCC	
*Bcl-2*	XM_027960877.2	F:TCTTTGAGTTCGGAGGGGTC	162
		R:GGCCATACAGCTCCACAAAG	
*BAK*	XM_015102660.4	F:GTCTTCCGCAGCTACGTCTT R:CGGTTGATGTCATCCCCGAT	161
*PCNA*	XM_004014340.5	F:CTTGGTGCAGCTAACCCTTC	161
		R:CCAAGGTGTCCGCATTATCT	
*TGF-β1*	NM_001009400.2	F:GAGCCCTGGACACCAACTAC	161
		R:GCTCCAGATGTAGGGACAGG	
		R:ACTTCTCCTTCAGGGCATCAC	
*WNT5A*	XM_004018630.5	F:AGGGCAATGTCTTCCAAGTTCTTC R:GTTATTCATACCTAGCGACCACCAAG	105
*GAPDH*	NM_001190390.1	F:GGTCGGAGTGAACGGATTT	175
		R:CTCTGCCTTGACTGTGCC	

## Data Availability

The datasets used and analyzed during this current study are available from the corresponding author upon reasonable request.

## Supplementary Materials

The following supporting information can be downloaded at www.mdpi.com/article/10.3390/ijms26051961/s1.
