# Supplementary material for: TGF-β1 Mediates Novel-m0297-5p Targeting WNT5A to Participate in the Proliferation of Ovarian Granulosa Cells in Small-Tailed Han Sheep
Source: Int J Mol Sci. 2025 Feb 24;26(5):1961. doi: 10.3390/ijms26051961 (PMC11901034; doi:10.3390/ijms26051961)
Supplement: Supplementary file 1 [file ijms-26-01961-s001.zip › ijms-3388113-supplementary.pdf]

Supplementary Information

Figure S1. (a) Homology comparison of TGF-β1 proprotein preproprotein between human and sheep.; (b) The homology of amino acid sequences was compared by DNAMAN 8.0

(a)

	Description	Scientific Name	Max Score	Total Score	Query Cover	E value	Per. Ident	Acc. Len	Accession
☑	transforming growth factor beta-1 proprotein preproprotein [Homo sapiens]	Homo sapiens	683	683	94%	0.0	94.28%	390	NP_000651.3

(b)

```
NP_000651.3_transforming_growthin_preproprotein_[Homo_sapiens]o                      V      A                  40
NP_001009400.1_transforming_grooprotein_precursor_[Ovis_aries]e                      M      V                  40
Consensus                                                          mppsglrllplllpllwll ltpgrp aglstcktidmel

NP_000651.3_transforming_growthin_preproprotein_[Homo_sapiens]o        R                     E         V        80
NP_001009400.1_transforming_grooprotein_precursor_[Ovis_aries]e        G                     D         I        80
Consensus                                                          vkrk ieairgqilsklrlasppsqg vppgplpea lal

NP_000651.3_transforming_growthin_preproprotein_[Homo_sapiens]o                  P                    TH E     120
NP_001009400.1_transforming_grooprotein_precursor_[Ovis_aries]e                  T                    YG K     120
Consensus                                                          ynstrdrvagesae epepeadyyakevtrvlmve  n i

NP_000651.3_transforming_growthin_preproprotein_[Homo_sapiens]o       F Q T                         EL         160
NP_001009400.1_transforming_grooprotein_precursor_[Ovis_aries]e       M S S                         DV         160
Consensus                                                          ydk k s hsiymffntselreavpepvllsra  rllrl

NP_000651.3_transforming_growthin_preproprotein_[Homo_sapiens]o                                                200
NP_001009400.1_transforming_grooprotein_precursor_[Ovis_aries]e                                                200
Consensus                                                          klkveqhvelyqkysnnswrylsnrllapsdspewlsfdv

NP_000651.3_transforming_growthin_preproprotein_[Homo_sapiens]o            SRGG               R           T    240
NP_001009400.1_transforming_grooprotein_precursor_[Ovis_aries]e            THRE               K           S    240
Consensus                                                          tgvvrqwl    eiegfrlsahcscds dntlqvdingf

NP_000651.3_transforming_growthin_preproprotein_[Homo_sapiens]o    T                              Q            280
NP_001009400.1_transforming_grooprotein_precursor_[Ovis_aries]e    S                              H            280
Consensus                                                           grrgdlatihgmnrpflllmatpleraqhl ssrhrral

NP_000651.3_transforming_growthin_preproprotein_[Homo_sapiens]o                                                320
NP_001009400.1_transforming_grooprotein_precursor_[Ovis_aries]e                                                320
Consensus                                                          dtnycfsstekncc vrqlyidfrkdlgwkwihepkgyhan

NP_000651.3_transforming_growthin_preproprotein_[Homo_sapiens]o                                                360
NP_001009400.1_transforming_grooprotein_precursor_[Ovis_aries]e                                                360
Consensus                                                          fclgpcpyiwsldtqyskvlalynqhnpgasaapccvpqa

NP_000651.3_transforming_growthin_preproprotein_[Homo_sapiens]o                                                389
NP_001009400.1_transforming_grooprotein_precursor_[Ovis_aries]e                                                389
Consensus                                                          leplpivyyvgrkpkveqlsnmivrsckc
```
